# Combination of RTS,S and Pfs25-IMX313 Induces a Functional Antibody Response Against Malaria Infection and Transmission in Mice

**DOI:** 10.3389/fimmu.2018.02780

**Published:** 2018-12-04

**Authors:** Florian Brod, Kazutoyo Miura, Iona Taylor, Yuanyuan Li, Arianna Marini, Ahmed M. Salman, Alexandra J. Spencer, Carole A. Long, Sumi Biswas

**Affiliations:** ^1^Jenner Institute, University of Oxford, Oxford, United Kingdom; ^2^Laboratory of Malaria and Vector Research, National Institute of Allergy and Infectious Disease, National Institutes of Health, Rockville, MD, United States

**Keywords:** malaria, vaccine, transmission blocking, pre-erythrocytic, synergy

## Abstract

The last two decades saw a dramatic reduction in malaria incidence rates, but this decrease has been stalling recently, indicating control measures are starting to fail. An effective vaccine, particularly one with a marked effect on disease transmission, would undoubtedly be an invaluable tool for efforts to control and eliminate malaria. RTS,S/AS01, the most advanced malaria vaccine to date, targets the parasite before it invades the liver and has the potential to prevent malaria disease as well as transmission by preventing blood stage infection and therefore gametocytogenesis. Unfortunately efficacy in a phase III clinical trial was limited and it is widely believed that a malaria vaccine needed to contain multiple antigens from different life-cycle stages to have a realistic chance of success. A recent study in mice has shown that partially efficacious interventions targeting the pre-erythrocytic and the sexual lifecycle stage synergise in eliminating malaria from a population over multiple generations. Hence, the combination of RTS,S/AS01 with a transmission blocking vaccine (TBV) is highly appealing as a pragmatic and powerful way to increase vaccine efficacy. Here we demonstrate that combining Pfs25-IMX313, one of the TBV candidates currently in clinical development, with RTS,S/AS01 readily induces a functional immune response against both antigens in outbred CD1 mice. Formulation of Pfs25-IMX313 in AS01 significantly increased antibody titres when compared to formulation in Alhydrogel, resulting in improved transmission reducing activity in standard membrane feeding assays (SMFA). Upon co-formulation of Pfs25-IMX313 with RTS,S/AS01, the immunogenicity of both vaccines was maintained, and functional assessment of the induced antibody response by SMFA and inhibition of sporozoite invasion assay (ISI) showed no reduction in biological activity against parasites of both lifecycle stages. Should this findings be translatable to human vaccination this could greatly aid efforts to eliminate and eventually eradicate malaria.

## Introduction

Malaria is caused by parasites of the genus *Plasmodium*, and it is one of the world's oldest and deadliest diseases. According to estimates in the World Health Organisation (WHO) world malaria report 2017 (1) the global number of malaria deaths reached 445,000 in 2016, and there were a total of 216 million malaria cases, an increase of 5 million cases over 2015. This is the first time since 2010 that case numbers increased with respect to the estimates of the previous year indicating that current control measures, which helped to drastically reduce malaria prevalence over the last decade are starting to fail and novel interventions are necessary if malaria eradication is to be achieved (2).

Vaccines are undoubtedly one of the most successful public heath interventions of all time, and played an essential role in the control or eradication of a number of diseases. An efficacious vaccine would be of immense value to all efforts to contain and eventually eradicate malaria. As outlined in the malaria vaccine technology (MVT) roadmap, as well as the malERA research agenda for malaria eradication (3, 4), an ideal malaria vaccine would have two characteristics; it would protect a vaccinated individual from illness as well as have an impact on malaria transmission (5). *Plasmodium* parasites have an exquisitely complex lifecycle with obligate developmental steps in the human host as well as in the mosquito vector. Roughly, it can be broken down in three different stages, the pre-erythrocytic stage, the erythrocytic stage or asexual blood stage and the sexual or sporogonic stage. Vaccines with an impact on malaria transmission are often referred to as VIMTs (vaccines that interrupt malaria transmission), and include classic TBVs that target the sexual life-cycle stage of the parasite in the mosquito vector as well as pre-erythrocytic vaccines (PEVs) that prevent blood stage infection and therefore gametocytogenesis. The impact of vaccines targeting the asexual blood stage, which also is the target of naturally acquired immunity, is likely to be more limited. The most advanced malaria vaccine candidate to date RTS,S/AS01 targets the pre-erythrocytic stage antigen circumsporozoite protein (CSP) of *P. falciparum*, the causative agent of the most severe form of human malaria, with the aim to interrupt the parasite's lifecycle before the establishment of liver stage infection. It is the only malaria vaccine to have completed Phase III clinical trials (6–9) and to have obtained a positive scientific opinion from the European Medicines Agency (EMA) (10). As RTS,S/AS01 targets a stage in the lifecycle before the emergence of symptomatic and transmissible stages, it has the potential to prevent disease as well as transmission. However, efficacy of RTS,S/AS01 in the recently concluded Phase III clinical trial, stayed below the targets formulated in the MVT roadmap (8, 9).

One way to build on the substantial efforts that led to development of RTS,S/AS01 is to combine it with other malaria vaccines currently in development, and indeed consensus is now that a vaccine containing multiple antigens from different life-cycle stages has the most realistic chance of success (11, 12). A recent study using two monoclonal antibodies targeting CSP and the sexual stage antigen Pfs25 at partially efficacious concentrations showed that pre-erythrocytic and sexual stage interventions synergise over multiple generations as the efficacy of PEVs is sensitively dependent on the sporozoite load of an infectious mosquito and TBVs can reduce the number of oocysts that develop in the mosquito midgut and therefore reduce the number of sporozoites that reach the mosquito's salivary gland (13). Therefore, combination of RTS,S/AS01 with a TBV, targeting the sexual stage of the parasite, is particularly attractive. Furthermore, addition of RTS,S/AS01 to a TBV could overcome one of the perceived drawbacks of TBVs, which is the absence of a direct protective effect on the vaccinee, while addition of a TBV to RTS,S/AS01 could increase the effect RTS,S has on malaria transmission, as well as reduce the emergence of escape mutants.

The most advanced candidate antigen for a transmission blocking malaria vaccine is Pfs25. It has been extensively tested in mouse models where immunization with Pfs25 containing vaccines can induce an antibody response that completely blocks parasite transmission to mosquitoes (14). Pfs25, as well as its *P. vivax* ortholog Pvs25, has been tested in clinical trials (15, 16) making it, alongside Pfs230, one of only two *P. falciparum* transmission blocking antigens to be tested in humans. This showed that antibodies against Pfs25 reduce malaria transmission in SMFA (15, 17) and that the transmission reducing activity (TRA) is correlated with the induced antibody titres. Unfortunately repeated immunizations resulted in above 50% TRA in only 9 out of 11 volunteers, and this was reduced to 2 out of 11 6 weeks later (17). Similar observations were made in early clinical trials with vaccines targeting CSP, where limited vaccine efficacy correlated with a moderate antibody response. This triggered the research and evaluation of improved vaccine candidates which eventually led to the development of RTS,S/AS01 (18–20). Consequently a number of strategies have been applied to increase the antibody response induced by vaccines targeting Pfs25 (14). These included conjugating Pfs25 to the exoprotein A of *Pseudomonas aeruginosa* (EPA), which substantially increase antibody titres against Pfs25 in mice (21), but clinical trials using Pfs25-EPA formulated in Allhydrogel found only limited and short lived serum transmission blocking activity in vacinees (16, 17). The shortcoming of Pfs25-EPA/Allhydrogel in clinical trials suggests that further antigen/adjuvant combinations need to be assessed to improve TRA of vaccines targeting Pfs25. One alternative antigen, currently under clinical development, is generated by multimerisation of Pfs25 by fusion to the nanoparticle platform IMX313 (Pfs25-IMX313). This resulted in increased antibody titres and TRA in mice after immunization, either when encoded in viral vectors or as protein in adjuvant formulations (22). Furthermore, RTS,S, the antigenic component of the RTS,S/AS01 vaccine, is delivered with a highly potent adjuvant (AS01) specifically developed to induce very high antibody responses. In this study we show that formulation of Pfs25-IMX313 with AS01 increases antibody responses against Pfs25 in mice. We also show that RTS,S and Pfs25-IMX313 can be combined without any immunological interference in mice, maintaining the level of antibody immunogenicity and functional activity induced by the individual vaccines. This is the first proof of concept of the compatibility of combining RTS,S with a TBV, which could be of great value, particularly in local malaria eradication campaigns.

## Results

### AS01 Increases Induced Antibody Titres After Immunization With Pfs25-IMX313

**Figure 1:**
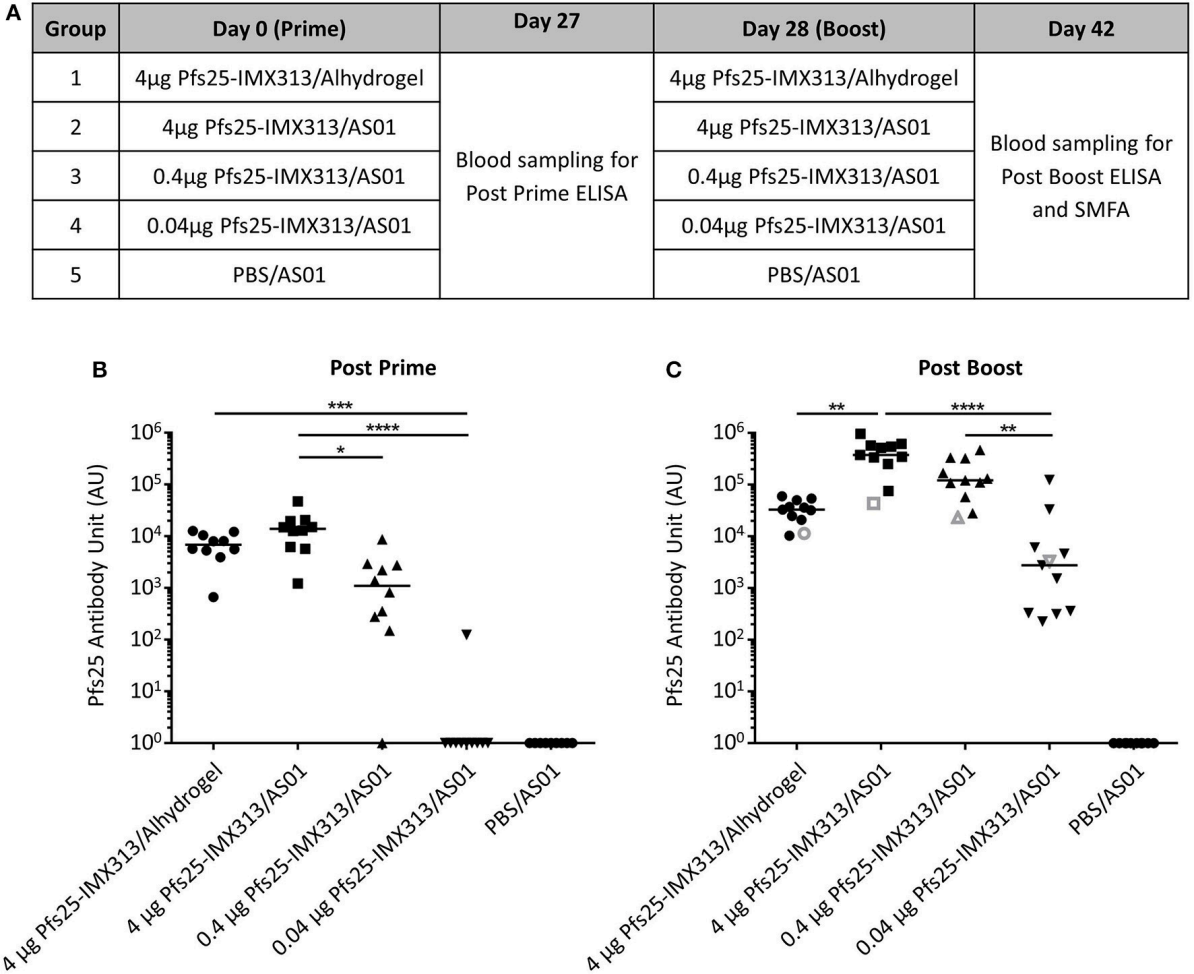
Immunogenicity of Pfs25-IMX313 in AlhydrogelFigure or AS01. **(A)** CD1 mice (*n* = 10) were vaccinated twice with 4 μg Pfs25-IMX313 in Alhydrogel or with decreasing doses (4 μg, 0.4 μg and 0.04 μg) of Pfs25-IMX313 in AS01. BC Anti-Pfs25 IgG titres were measured by ELISA at 4 weeks post prime **(B)** and 2 weeks post boost **(C)**. Empty gray shapes show the antibody units of a 1 mg/ml dilution of purified IgG. Data points show antibody titres in individual mice, lines show the median. ^*^p ≤ 0.05, ^**^p ≤ 0.005, ^***^p ≤ 0.001, ^****^p ≤ 0.0001, Dunn's multiple comparison test.

Pfs25-IMX313 was purified and expressed as previously described (22), formulated in adjuvant and administered to mice, to assess whether the antibody response induced after vaccination with Pfs25-IMX313 could be improved further by using AS01 instead of Alhydrogel as the adjuvant. Three doses of Pfs25-IMX313 were tested in order to measure if there was a dose sparing effect. Ten CD-1 mice per group were immunized on day 0 and day 28 (Figure 1A). Blood samples were collected at day 27 and day 42 and anti-Pfs25 IgG titres assessed by ELISA (Figures 1B,C). On day 27 after primary immunization, only vaccine formulations containing 4 μg of Pfs25-IMX313 induced a detectable antibody response in all mice. The next lower dose, 0.4 μg of Pfs25-IMX313 in AS01 induced a detectable immune response in 9 out of 10 mice, but the induced antibody titres were significantly lower than those induced by 4 μg of Pfs25-IMX313 in AS01 (*p* = 0.0118, Dunn's multiple comparison test). The lowest dose tested, 0.04 μg of Pfs25-IMX313 in AS01, induced a detectable anti-Pfs25 IgG response only in 1 out of 10 mice, and the induced antibody titres were significantly lower than those induced by both formulations containing 4 μg of Pfs25-IMX313 (4 μg of Pfs25-IMX313 in AS01 *p* < 0.0001, 4 μg of Pfs25-IMX313 in Alhydrogel *p* = 0.0006, Dunn's multiple comparison test).

After boosting, all vaccine formulations induced a detectable immune response in all mice on day 42, while no detectable immune response was induced in the mice immunized with PBS/AS01. The two highest doses of Pfs25-IMX313 in AS01 induced the highest anti-Pfs25 IgG titres. 4 μg of Pfs25-IMX313 in Alhydrogel induced significantly lower titres than the same vaccine dose in AS01 (*p* = 0.0014). Titres induced by 0.04 μg of Pfs25-IMX313 in AS01 were significantly lower than those induced by the two higher doses in the same adjuvant (4 μg of Pfs25-IMX313 in AS01 *p* < 0.0001, 0.4 μg of Pfs25-IMX313 in AS01 *p* = 0.004, Dunn's multiple comparison test), but not than those induced by the 100-fold higher dose of 4 μg of Pfs25-IMX313 in Alhydrogel. Formulation of Pfs25-IMX313 in AS01 therefore clearly increases the vaccine's immunogenicity, but while there is a dose sparing effect when compared to formulation in Alhydrogel, the highest antibody titres are achieved by immunization with the highest tested dose.

### Antibodies Induced by Pfs25-IMX313/AS01 are Functionally Active

**Figure 2:**
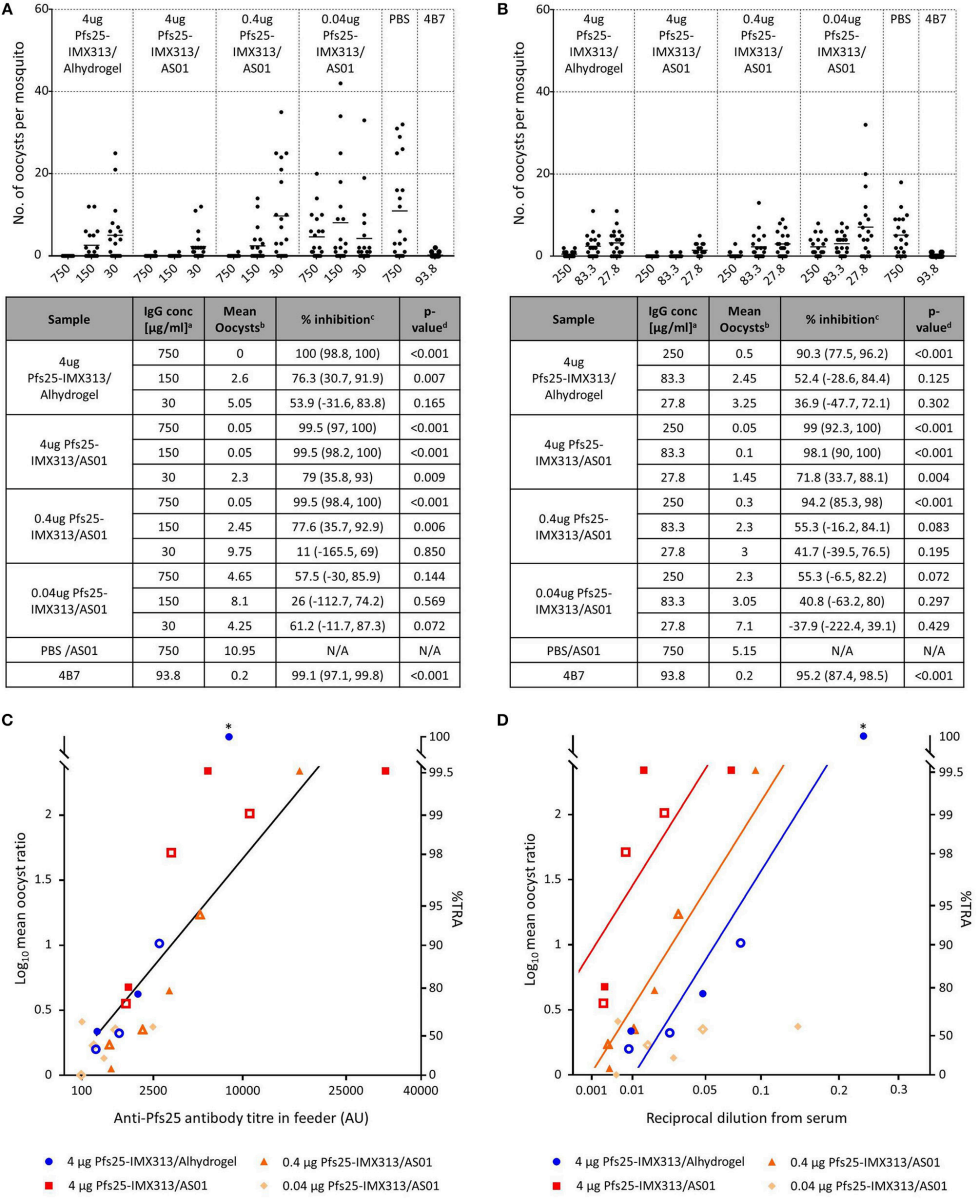
Transmission blocking activity of anti Pfs25-IgG induced by immunization with Pfs25-IMX313 in Alhydrogel or AS01. **(A,B)** Total IgG, purified from pooled serum of each group (2 weeks post boost) was tested in SMFA in two independent experiments, one using total IgG concentrations of 750, 150 and 30 μg/ml **(A)** and one using total IgG concentrations of 250, 83.3, and 27.7 μg/ml **(B)**. The transmission blocking monoclonal anti-Pfs25 antibody 4B7 was used as a positive control. Data points represent the number of oocysts in individual mosquitoes and the lines show the arithmetic mean. X-axis values are μg/ml total IgG in the assay. The results of the two feeds are summarized in the tables. ^a^IgG concentration (μg/ml) in feeder. ^b^Arithmetic mean of oocyst intensity from 20 mosquitoes. ^c^Percent inhibition of mean oocyst intensity and the 95% confidence interval (95% CI). ^d^Two-sided *p*-values testing whether % inhibition is significantly different from zero. **(C,D)** The quality of the anti-Pfs25-IgG used in the SMFA was assessed by linear two regression analyses, correlating the Log10-transformed ratios of mean oocyst counts in control and test samples (log-mean ratio, LMR) with the anti-Pfs25 specific IgG level in the feeder **(C)** or the reciprocal dilution factor from the original serum pool **(D)**. LMR is plotted on the left y-axis. The right y-axis shows the correspondent %TRA. Values on the x-axis are plotted on a square root scale. The black line shows the shared linear fit for IgG from groups of mice immunized with 4 μg Pfs25-IMX313 in Alhydrogel or with 4 μg and 0.4 μg of Pfs25-IMX313 in AS01. Colored lines show linear fits for individual groups. Filled and unfilled shapes show data points from two independent SMFA experiments. Data points marked with an asterisk were excluded from the analysis as they showed 100% TRA (upper plateau level of dose response).

Functional activity of the induced antibodies after the second immunization was assessed by SMFA. Live mosquitoes were fed with a mixture of *P. falciparum* gametocyte-infected blood and purified IgG from each group, and the number of oocysts which developed in the midgut was determined after dissection (Figure 2). Functional efficacy was calculated as transmission reducing activity (TRA), the reduction in the number of oocysts compared to the negative control group. In a first feed, total IgG concentrations of 750, 150, and 30 μg/ml were tested. All groups apart from the one that received 0.04 μg Pfs25-IMX313/AS01 induced statistically significant TRA when compared to the negative control at the two highest IgG concentrations, but only 4 μg Pfs25-IMX313/AS01 induced statistically significant TRA at 30 μg/ml. To further dissect differences between groups at lower IgG concentrations a second feed was performed using 250, 83.3, and 27.8 μg/ml IgG. This confirmed results from the first feed with only 4 μg Pfs25-IMX313/AS01 inducing statistically significant TRA at all three IgG concentrations, suggesting the high antibody titres observed in this group correlate with improved TRA.

### Adjuvant and Vaccine Dose do Not Affect Antibody Quality

As a means to assess whether differences in TRA between groups were caused by a qualitative or a quantitative difference in the antibody response, the SMFA results were correlated with the amount of Pfs25 specific IgG in the feeder (Figure 2C). Datasets for 4 μg Pfs25-IMX313/Alhydrogel, 4 μg Pfs25-IMX313/AS01 and 0.4 μg Pfs25-IMX313/AS01 were analyzed by linear regression. The 0.04 μg Pfs25-IMX313/AS01 group was excluded from the analyses due to the absence of any statistically significant TRA in this group. To determine the transmission reducing efficacy per anti-Pfs25 AU, anti-Pfs25 AU and vaccine groups were used as explanatory variables in a multiple linear regression analysis. The overall fit to the linear regression model was *R*^2^ = 0.78, and anti-Pfs25 AU (*p* < 0.001) but not vaccine groups (*p* = 0.1625) showed a significant effect. TRA in all three groups is therefore dependent on the amount of IgG used in SMFA but not on the dose or adjuvant the vaccine was administered in, suggesting that there is no qualitative difference between the antibody responses induced by the different vaccine regimens. This, as well as the differences in the induced antibody titres measured by ELISA (Figure 1C), suggests that the difference in induced TRA is due to an quantitative, not a qualitative difference in the antibody response. To confirm this, a similar linear regression analysis as before was performed using the reciprocal dilution factor of the serum pool as one of the explanatory variables, instead of anti-Pfs25 AU (Figure 2D). This allows an indirect analysis of the antibody response's efficacy in the undiluted mouse serum from each group, therefore providing a combined assessment of antibody quantity and quality. The overall fit to the linear regression model was *R*^2^ = 0.68 and the serum dilution factor (*p* < 0.001) as well as the vaccine group (*p* = 0.0001) showed significant effects. When three groups were compared, 4 μg Pfs25-IMX313/Alhydrogel and 0.4 μg Pfs25-IMX313/AS01, which had both induced a similar antibody titres also appeared to have induced a similarly efficacious response (*p* = 0.0924), while both of these vaccine formulations induced a significantly less efficacious antibody response than 4 μg Pfs25-IMX313/AS01 (*p* < 0.001 and *p* = 0.0026, respectively), which had induced a significantly higher antibody titer. This analysis therefore confirms the observation from the antibody quality analysis, that the observed difference in SMFA and the superior performance of the 4 μg Pfs25-IMX313/AS01 vaccine formulation is due to a quantitative but not a qualitative difference in the induced antibody response.

### Pfs25-IMX313 can be Combined With RTS,S Without Immunological Interference

**Figure 3:**
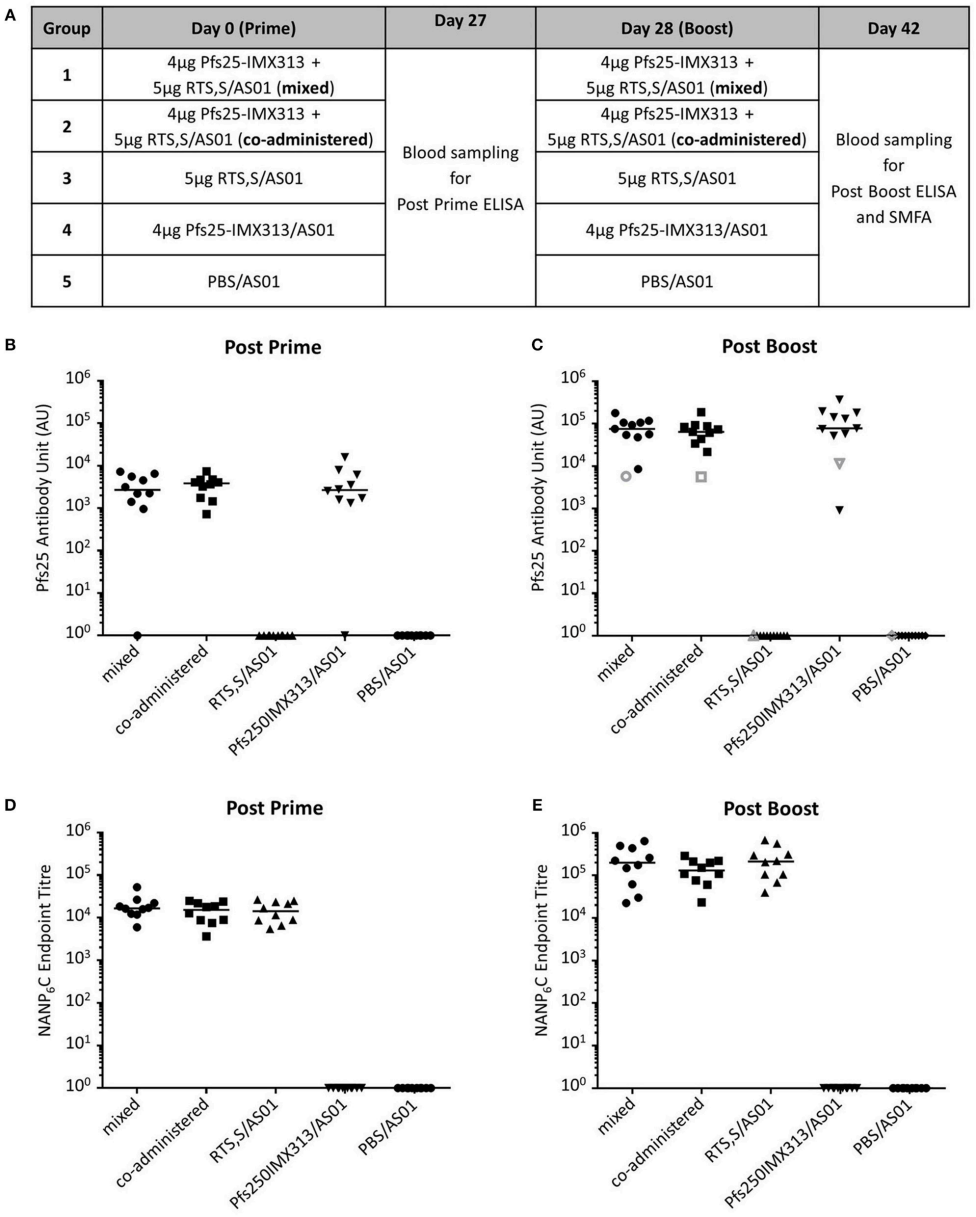
Immunogenicity of Pfs25-IMX313 and RTS,S alone or when combined. **(A)** CD1 mice (*n* = 10) were vaccinated twice with (1) a mixture of 4 μg Pfs25-IMX313 and 5 μg RTS,S/AS01, (2) 4 μg Pfs25-IMX313/AS01 and 5 μg RTS,S/AS01, administered separately in individual hind legs, (3) 5 μg RTS,S/AS01 and (4) 4 μg Pfs25-IMX313/AS01. A control group (5) was immunized with PBS + AS01. **(B,C)** Anti-Pfs25 IgG titres were measured by ELISA at 4 weeks post prime **(B)** and 2 weeks post boost **(C)**. Empty shapes show the antibody units of a 1 mg/ml dilution of purified IgG. **(D,E)** Anti-NANP6C IgG endpoint titres were measured by ELISA at 4 weeks post prime **(D)** and 2 weeks post boost **(E)**. Data points show antibody titres in individual mice, lines show the median.

Having established that AS01 increases the immunogenicity and efficacy of Pfs25-IMX313 as well as determined an optimal dose, we assessed whether Pfs25-IMX313 could be combined with RTS,S/AS01 as a multi-antigen, multi-stage malaria vaccine. Ten CD1 mice per group were immunized on day 0 and day 28 (Figure 3A). The mixed vaccine group was immunized with RTS,S/AS01 and Pfs25-IMX313 combined into a single injection, while the co-administered group received both vaccines separately into the left and the right hind leg, respectively. In the latter group, each injection was formulated with only half a dose of AS01, to keep the total amount of administered adjuvant constant. Blood samples were collected at day 27 and day 42 and anti-Pfs25 and anti-CSP IgG titres were assessed by ELISA (Figure 3). All Pfs25-IMX313 containing vaccine formulations induced strong anti-Pfs25 antibody responses and all RTS,S containing vaccine formulations induced strong anti-CSP antibody responses. Mixing or co-administration of the two vaccines did not result in any significant reduction in antibody titres when compared to the groups that had received Pfs25-IMX313/AS01 or RTS,S/AS01 alone, suggesting that there is no immune competition between the two vaccines.

### Combination of Pfs25-IMX313 With RTS,S Does not Reduce the Functional Activity of Anti-Pfs25 IgG

**Figure 4:**
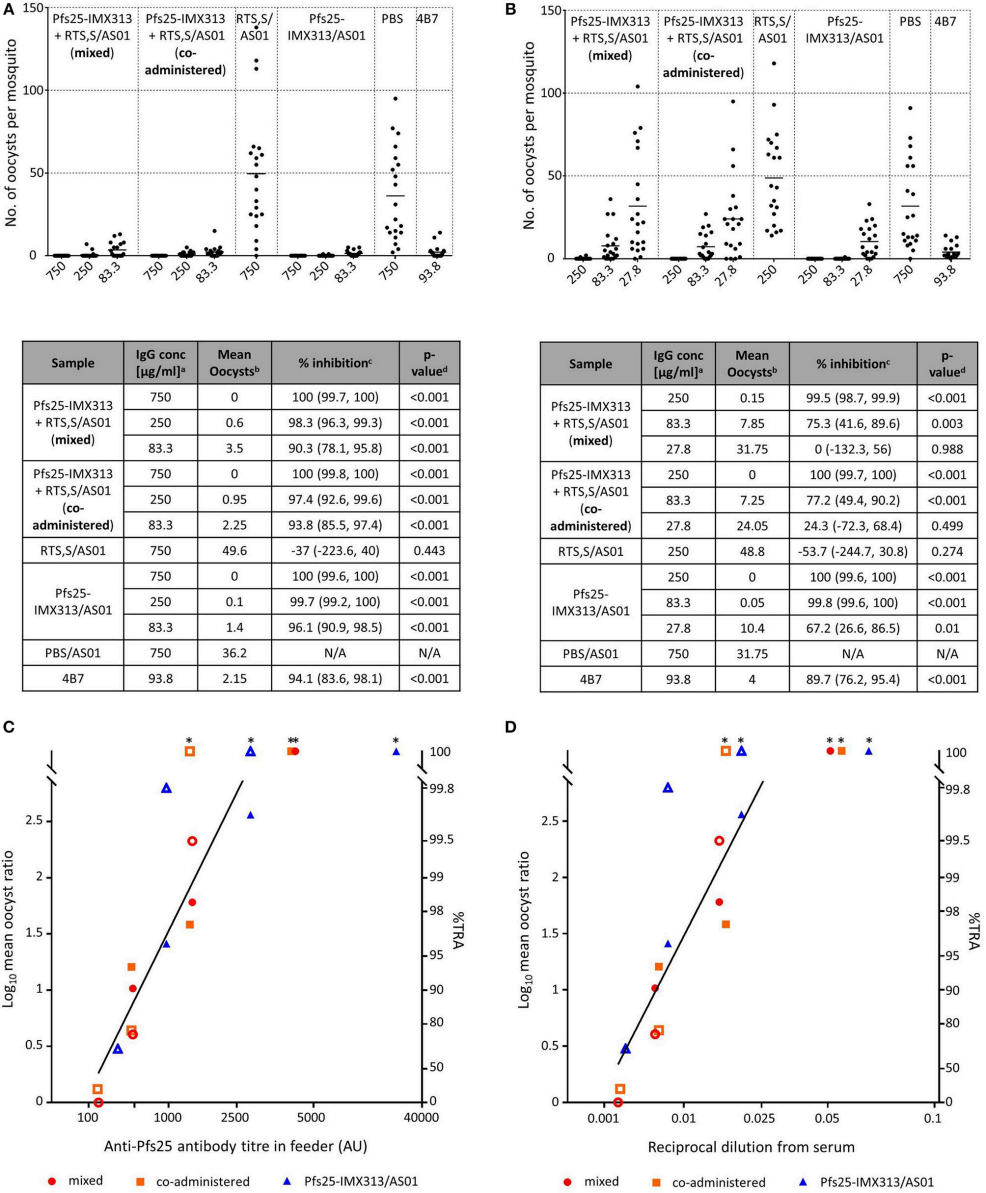
Transmission blocking activity of anti Pfs25-IgG induced by immunization with Pfs25-IMX313 alone or in combination with RTS,S. **(A,B)** Total IgG, purified from pooled serum of each group (2 weeks post boost) was tested in SMFA in two independent experiments, one using total IgG concentrations of 750, 250 and 83.3 μg/ml **(A)** and one using total IgG concentrations of 250, 83.3, and 27.7 μg/ml. The transmission blocking monoclonal anti-Pfs25 antibody 4B7 was used as a positive control. Data points represent the number of oocysts in individual mosquitoes and the lines show the arithmetic mean. X-axis values are μg/ml total IgG in the assay. The results of the two feeds are summarized in the tables. ^a^IgG concentration (μg/ml) in feeder. ^b^Arithmetic mean of oocyst intensity from 20 mosquitoes. ^c^Percent inhibition of mean oocyst intensity and the 95% confidence interval (95% CI). ^d^Two-sided *p*-values testing whether % inhibition is significantly different from zero. **(C,D)** The quality of the anti-Pfs25-IgG used in the SMFA was assessed by linear two regression analyses, correlating the Log10-transformed ratios of mean oocyst counts in control and test samples (log-mean ratio, LMR) with the anti-Pfs25 specific IgG level in the feeder **(C)** or the reciprocal dilution factor from the original serum pool **(D)**. LMR is plotted on the left y-axis. The right y-axis shows the correspondent %TRA. Values on the x-axis are plotted on a square root scale. The black line shows the shared linear fit for IgG from all groups. Filled and unfilled shapes show data points from the two independent SMFA experiments. Data points marked with an asterisk were excluded from the analysis as they showed 100% TRA (upper plateau level of dose response).

Functional activity of anti-Pfs25 IgG was assessed by SMFA (Figure 4). In a first feed, IgGs from all Pfs25-IMX313 containing groups were tested at 750, 250, and 83.3 μg/ml, groups that had received RTS,S/AS01 or PBS/AS01 were tested at 750 μg/ml only. Significant TRA was observed for all groups that received Pfs25-IMX313 at all the tested concentrations. Immunization with RTS,S/AS01 had no statistically significant effect on oocyst intensity. In a second feed we therefore tested lower IgG concentrations (250, 83.3, and 27.8 μg/ml) to assess for differences between groups at these lower concentrations. As before, at IgG concentrations of 250 and 83.3 μg/ml all groups that had received Pfs25-IMX313 showed significant TRA, while immunization with RTS,S/AS01 had no statistically significant effect when compared to immunization with PBS/AS01. At the lowest tested concentration of 27.8 μg/ml, only the Pfs25-IMX313 alone group showed significant TRA. Correlation of antibody titres in the feeder with the SMFA results, however, did not indicate a difference in antibody quality between combination and individually administered vaccine (Figure 4C). The overall fit to the linear regression model was *R*^2^ = 0.68, and again anti-Pfs25 AU (*p* = 0.0012) but not vaccine groups (*p* = 0.7322) showed a significant effect. Correlation of SMFA results with reciprocal serum dilutions furthermore confirmed the quantification of the antibody response by ELISA (Figure 3C) in indicating there was no quantitative difference in the IgG response between the groups (*R*^2^ = 0.69, serum dilution factor: *p* = 0.001, group: *p* = 0.1275). The observed difference in SMFA is therefore most likely due to a higher concentration of Pfs25-specific IgG in the purified total IgG from the Pfs25-IMX313 alone group, than in the total IgG purified from the groups that received both vaccines (log_10_ antibody titer of 1 mg/ml purified IgG: mixed = 3.76, co-administered: 3.75, Pfs25-IMX313 alone: 4.06). This is however not reflective of the amount of Pfs25-specific IgG in serum of immunized mice, which was similar in all three groups (arithmetic mean log_10_ antibody titer: mixed = 4.83 AU, co-administered = 4.81 AU, Pfs25-IMX313 alone = 4.86 AU).

### Combination of RTS,S With Pfs25-IMX313 Does not Reduce the Functional Activity of Induced Anti-CSP Antibodies

**Figure 5:**
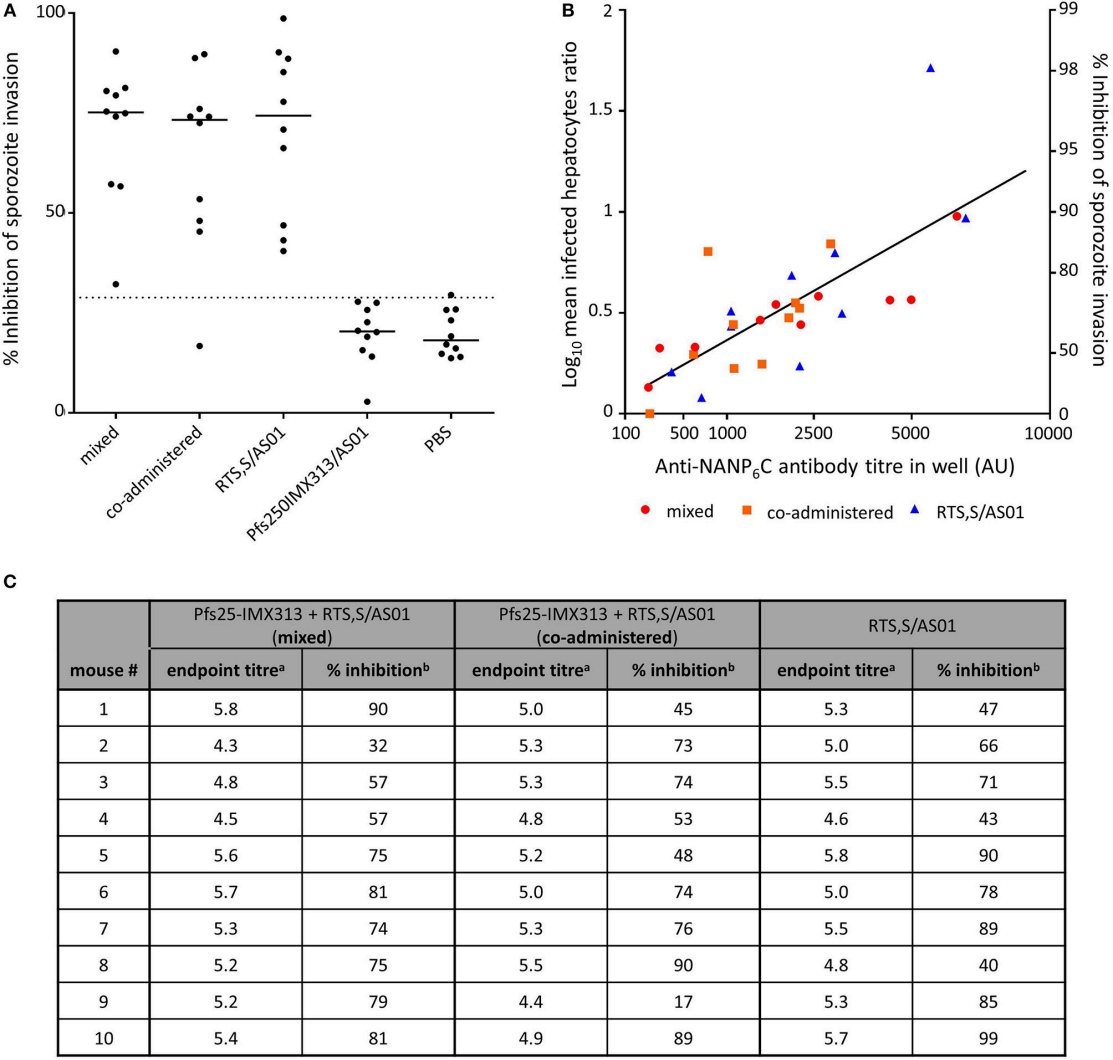
Inhibition of sporozoite invasion by anti CSP-serum induced by immunization with RTS,S alone or in combination with Pfs25-IMX313. **(A)** Serum from individual mice (2 weeks post boost) was mixed with *P. berghei* sporozoites from mosquito salivary gland dissections, and the inhibition of sporozoite invasion was assessed. Data points show inhibition of sporozoite invasion by serum from individual mice, lines show the median, the dotted line shows the limit of sensitivity. **(B)** The quality of anti-CSP serum induced in individual mice was assessed by linear regression analysis, correlating the anti-CSP IgG endpoint titres in the culture medium with the log-transformed ratios of infected hepatocytes in control and test samples (log-mean ratio, LMR). LMR is plotted on the left y-axis. The right y-axis shows the correspondent %ISI. Values on the x-axis are plotted on a square root scale. The black line shows the shared linear fit for IgG from all groups. **(C)** The induced anti-NANP endpoint titres and the correlating %ISI in individual mice are detailed in the table. ^a^Log10-transformed NANP6C endpoint titres as determined by ELISA. ^b^% Inhibition of sporozoite invasion by 1% serum in culture medium.

Functional activity of anti-CSP antibodies was assessed by ISI assay using *P.berghei* sporozoites expressing PfCSP under the PbCSP promoter (Figure 5). Sporozoites were obtained from the salivary glands of infected *A. stephensi* mosquitoes and added to Huh7 hepatoma cells in the presence of test and control serum. At 1% serum concentration inhibition of sporozoite invasion is detectable in all groups that received RTS,S. There is no statistically significant difference between groups immunized with RTS,S/AS01 alone or mixed or co-administered with Pfs25-IMX313, but all groups that received RTS,S inhibit sporozoite invasion above the assay's limit of sensitivity and inhibition was significantly higher than in the group that had been immunized with Pfs25-IMX313 (mixed *p* = 0.0011, co-administered *p* = 0.0154, RTS,S alone *p* = 0.0015, Dunn's multiple comparison test). To further characterize the anti-CSP antibody response induced by the different vaccine formulations containing RTS,S the log transformed mean ratio of infected hepatocytes in the vaccinated and PBS control groups was plotted against the square root of the anti-CSP endpoint titer in the hepatocyte growth medium, as had been done to analyse SMFA results. For all vaccine groups there was a significant correlation between increased LMR and higher anti-CSP AU (one-tailed Spearman correlation, mixed *r* = 0.9273, *p* = 0.0002, co-administered *r* = 0.612, *p* = 0.0334, RTS,S alone *r* = 0.8601, *p* = 0.0036. In a multiple linear regression analysis anti-CSP AU and vaccine groups were used as explanatory variables. The overall fit to the linear regression model was *R*^2^ = 0.55, and anti-CSP AU (*p* < 0.001) but not vaccine groups (*p* = 0.7913) showed a significant effect on the inhibition of sporozoite invasion, indicating that there is no loss of quality in the antibody response when RTS,S is combined with Pfs25-IMX313 rather than administered alone.

## Discussion

The modest efficacy of recent clinical trials using single antigen subunit vaccines support the development of multi-antigen vaccines targeting multiple stages of malaria. Mixing vaccines in a multi-component formulation is an easy process that does not require developing a new product. This means that it is possible to draw from previous experience in product development and clinical trials. This experience is particularly rich in the case of RTS,S the most advanced malaria vaccine candidate to date. Pfs25-IMX313 is a TBV candidate that we developed specifically to overcome the low antibody titres induced by vaccines targeting Pfs25 in past clinical trials (15, 17). Encoded in viral vectors it has now been tested in a clinical trial (ClinicalTrials.gov Identifier: NCT02532049), and it is currently being produced as a nanoparticle to GMP standards to be tested in clinical trials as a protein in adjuvant formulation, which in mice induced higher antibody titres than viral vectors (22). Here we showed that Pfs25-IMX313 can be formulated in AS01, which results in increased antibody titres when compared to formulation in Alhydrogel as well as increased TRA in SMFA. The functional activity of the induced antibodies per AU is similar for both adjuvants, but the overall stronger antibody response induced by Pfs25-IMX313/AS01 results in a substantially improved efficacy over Pfs25-IMX313/Alhydrogel.

This study also shows that RTS,S and Pfs25-IMX313 can be combined successfully maintaining both the antibody titres and functional activity of antibodies against CSP and Pfs25 compared to the individual vaccines. The perceived lower activity in SMFA of the combined vaccines is explained by the higher amount of antigen specific IgG in the total IgG preparation of the Pfs25-IMX313 alone group (Figure 3C), and not an inferior antibody quality in the groups that received both vaccines (Figure 4C). Quantification of anti-Pfs25 serum antibody titres by ELISA (Figure 3C) and correlation of TRA with reciprocal serum dilutions (Figure 4D) showed that a similar amount of anti-Pfs25 specific IgG had been generated in all three groups. However, in the groups that received RTS,S as well as Pfs25-IMX313 there is an additional strong antibody response against the CSP component in RTS,S. A fixed amount of total IgG from these groups will therefore contain a significant amount of anti-CSP antibodies not present in a total IgG preparation from mice that had been immunized with Pfs25-IMX313 alone, and hence a smaller amount of anti-Pfs25 specific IgG. A trend for increased oocyst intensity after immunization with RTS,S/AS01 (Figure 4) is not statistically significant, and most likely due to the high variability of the assay at lower inhibition range, rather than to an enhancing effect of RTS,S/AS01 on parasite transmission. This strongly suggests that there is no negative effect on the TRA induced by immunization with Pfs25-IMX313 when the vaccine is either mixed or co-administered with RTS,S/AS01.

The successful combination of the two vaccines contrasts with previous studies where the combination of different viral vectored malaria vaccines did result in strong immune interference (23, 24) and the combination of RTS,S/AS01 with a viral vector PEV (ChAd63/MVA ME-TRAP) failed to improve vaccine efficacy when compared to the two vaccines administered individually and reduced immunogenicity and efficacy against malaria (25). This success might be attributed to shared characteristics and modes of action of RTS,S/AS01 and Pfs25-IMX313. Both vaccines target antigens that are expressed with high abundance on the surface of the sporozoites and zygotes/ookinetes, respectively, both vaccines are produced in yeast and both vaccines use a particulate platform to array the antigen in order to induce a strong antibody response.

The combination of RTS,S/AS01 and Pfs25-IMX313, as we demonstrated here, is of particular interest, for combining a TBV with a vaccine that protects from clinical disease creates a vaccine which reduces disease as well as malaria transmission, as called for in the MVT roadmap and the malERA research agenda for malaria eradication (malERA 3; 5; malERA 4). Furthermore, a recent study in mice, using monoclonal antibodies against CSP and Pfs25, suggests that partially efficacious PEVs and TBVs could be synergistic within a population (13), as the protective efficacy of PEVs is negatively correlated with the parasite density within an infectious mosquito (26), which can be reduced through the presence of anti-Pfs25 antibodies in the human host (27). Finally, not being a target of naturally acquired immunity against malaria and therefore not under immune pressure, the sequence of Pfs25 is highly conserved (28). The selection of *P. falciparum* variants less affected by anti-CSP antibodies induced by RTS,S/AS01 can therefore likely be reduced through the presence of anti-Pfs25 which reduce the human-to-mosquito transmission of such escape variants. The advanced stage of RTS,S/AS01 and Pfs25-IMX313 as malaria vaccine candidates harbors the intriguing prospect of being able to test these hypothesis, with their potentially far reaching consequences for malaria control, in human clinical trials.

## Material and Methods

### Vaccines and Adjuvants

RTS,S and AS01 were provided by GSK. Pfs25-IMX313 was expressed and purified as described previously (22). In brief: Pfs25-IMX313 was expressed in *Pichia pastoris* under the control of the methanol inducible promoter AOX1. N-terminal fusion to the alpha-mating factor secretion signal sequence allowed secretion into the culture media. Following expression, nanoparticles were purified from the culture supernatant using a 5 ml CaptureSelect™ C-tag affinity matrix column.

### Immunizations

All animal experiments and procedures were performed according to the UK Animals (Scientific Procedures) Act Project License (PPL 30/2889) and approved by the Oxford University Local Ethical Review Committee. Age-matched female CD1 mice (Envigo, United Kingdom), housed in specific pathogen-free environments, were vaccinated with 50 μl of vaccine formulation in one leg intramuscularly (IM). Protein-in-Alhydrogel was formulated as follows: 85 μg Alhydrogel (Brenntag) per dose was mixed with TBS at room temperature for 15 min, antigen was then added and incubated for a further 60 min at room temperature. AS01E was supplied pre-mixed by GSK to be at 1-fold concentration upon mixing with an appropriate amount of antigen. Total protein per dose for each group is detailed in the tables in Figures 1, 3. Animals were immunized on days 0 and 28, and blood samples were collected on days 27 (4 weeks post prime) and 42 (2 weeks post boost). The samples were allowed to clot at 4°C overnight before centrifugation at 13,000 × *g* in a benchtop centrifuge and serum was collected for testing.

### ELISA

Standardized Pfs25 total IgG ELISAs were carried out as described previously (29). In brief, Nunc-Immuno Maxisorp 96 well plates (Thermo Scientific, UK) were coated with 1μg/ml Pfs25 in carbonate-bicarbonate coating buffer (Sigma Aldrich, UK) overnight at 4°C. Plates were washed with PBS-Tween and blocked with 5% milk in PBS-Tween. Sera were diluted to reach an OD405 in the linear range of the standard curve at the same time an internal control reaches an OD405 of 1. Samples were added in triplicates. An internal control was added in six replicates. A standard curve was added in duplicates, starting with a 1:1,000 dilution of reference sera diluted down the plate in 1:2 steps 10 times. Plates were incubated for 2 h at room temperature and then washed as before. Goat anti-mouse whole IgG conjugated to alkaline phosphatase (Sigma Aldrich, UK) was added for 1 h at room temperature. Following a final wash, plates were developed by adding p-nitrophenylphosphate at 1 mg/mL in diethanolamine buffer (Pierce, UK) and optical density (OD) was read at 405 nm. Antibody units (AU) were defined as the dilution of a serum sample at which it had the same OD as the internal control. AUs were extrapolated from the samples OD on the linear range of the standard curve.

NANP-repeat endpoint total IgG ELISAs were carried out as described previously (30). Nunc-Immuno Maxisorp 96 well plates (Thermo Scientific, United Kingdom) were coated with 2 μg/ml NANP6C peptide in carbonate-bicarbonate coating buffer (Sigma Aldrich, United Kingdom) overnight at 4°C. Plates were washed with PBS-Tween and blocked with 10% Casein Block (Thermo Scientific, United Kingdom). Sera were diluted at a starting concentration of 1:1,000, added in duplicate, and serially diluted 3-fold. Plates were incubated for 2 h at room temperature and then washed as before. Goat anti-mouse whole IgG conjugated to alkaline phosphatase (Sigma Aldrich, United Kingdom) was added for 1 h at room temperature. Following a final wash, plates were developed by adding p-nitrophenylphosphate at 1 mg/mL in diethanolamine buffer (Pierce, UK) and optical density (OD) was read at 405 nm. Serum antibody endpoint titres were taken as the x-axis intercept of the dilution curve at an absorbance value of 0.15. A monoclonal antibody against CSP (2A10) was included in each assay as a reference control.

### SMFA

The ability of vaccine-induced antibodies to block the development of *P. falciparum* NF54 strain oocysts in the mosquito midgut was evaluated by SMFA as described previously (31). Stage V gametocytes from a mature gametocyte culture were mixed with normal human serum and normal red blood cells to make a feeding mixture with 0.15–0.2% stage V gametocytemia. Purified IgG was added to these at the concentrations shown in the figures and then fed to 3–6 day old starved female *Anopheles stephensi* (SDA 500) via a parafilm® membrane. The mosquitoes were maintained for 8 days and then dissected to count the number of oocysts per midgut in 20 mosquitos. The transmission blocking, Pfs25 specific monoclonal antibody 4B7 was used as a positive control (31). Percent reduction in infection intensity was calculated relative to the respective control IgG tested in the same assay.

### Parasite Production

To produce parasites for the ISI assay, donor mice were intraperitoneally injected with a parasite bloodstock. Parasitaemia was monitored through blood sampling by tail tip amputation and thin film preparation. As soon as the parasitaemia exceeded 5%, the mouse was anesthetized by intra muscular injection of ketamine and placed on top of a pot containing approximately 50 female *A. stephensi* mosquitoes. Mosquitoes were allowed to feed through the mesh covering the pots for at least 10 min. Seven to ten days after the blood meal mosquitoes were allowed to feed on an uninfected mouse, as this had been shown to increase the number of parasites per mosquito. Twenty-one days after the initial feed salivary glands were dissected from the mosquitoes to obtain sporozoites.

### ISI

Functional activity of anti-sporozoite antibodies was assessed by ISI using transgenic *Plasmodium berghei* parasites expressing PfCSP under the PbCSP promoter, at the PbCSP locus, replacing the PbCSP gene, as well as GFP to allow detection of infected cells by flow cytometry (Salman, in preparation). This strain of transgenic parasites has been validated for use in ISI (32) and in murine malaria challenge models (33). One day prior to mosquito dissection for isolation of sporozoites, HuH7 cells were seeded in a 96 well plate at a concentration of 300,000 cells/ml in a volume of 100 μl/well, resulting in a concentration of 30,000 cells per well. One day later mosquitoes were dissected and salivary glands collected in ice cold RPMI. Sporozoites were released from salivary glands by tissue disruption using a pestle and mortar, and counted in a hemocytometer. Sera were diluted to double the required concentration in 120 μl complete RPMI. The medium was removed from the HuH7 cells seeded the day before, and 50 μl of diluted serum was added to the wells in duplicates. Sporozoites were then diluted to 300,000 sporozoites per ml in complete RPMI and 50 μl were added to each well, resulting in 15,000 sporozoites per well and a ratio of cells to sporozoites of 2:1. To aid sporozoite invasion of the cells, the plate was centrifuged at 500 × g for 5 min before incubation at 37°C and 5% CO_2_ overnight in a tissue culture incubator. After 24 h of incubation, cells were detached from the plate by removing medium from the plate and then adding 100 μl of TrypLE ™ Express (Gibco) to the wells and incubation for 5 min at 37°C and then transferred into cluster tubes. The empty wells were washed twice with 200 μl FACS buffer (PBS +0.5% BSA +0.05% Azide). Both washes were added to the cluster tubes. Cells were pelleted in the cluster tubes by centrifuging at 2,000 rpm for 2 min and the supernatant discarded. Cells were then resuspended in 80 μl FACS buffer. Directly before running cells on an LSR II flow cytometer (BD Biosciences), DAPI was added to the cells to a final concentration of 1 μg/ml, which allowed the separation of live from dead cells. Samples were acquired with a LSR II flow cytometer (BD Biosciences) using FACSDIVA software V 6.2 (BD Biosciences). *P*. *berghei* infected cells were identified by gating on viability and size, removing doublets and gating on GFP positive but PE (autofluorescene) negative cells (32).

### Statistical Analysis

Comparison of antibody titers and ISI results was performed using a Kruskal-Wallis test, which was followed up by a Dunn's multiple comparison test.

TRA was calculated from SMFA data, as 100 × [1- (mean number of oocysts in test/mean number of oocysts in control)] and 95% confidence intervals (95%CIs) of % inhibition in oocyst density from a single or multiple feeding experiments for each test antibody at each concentration were calculated using a zero inflation negative binomial model, as described previously (31).

ISI was calculated from cytometer acquisitions files (.fcs) using FlowJo.V 9.7.6 (Tree Star). The percentage of sporozoite inhibition was calculated as a reduction in the percentage of infected cells observed in untreated wells (average) compared to the percentage of infected cells observed in the presence of serum. The sensitivity limit of the assay was defined as the mean of the negative control + 1.645 standard deviations, i.e., 95% of observed values in the negative control, assuming a Gausian distribution.

A linear regression model was used to evaluate differences in functional activity among vaccine groups. The log_10_ transformed ratio of the mean oocyst count in control and test samples for SMFA, or the ratio of the percentage of viable, GFP positive hepatoma cells in control and test sample for ISI, was the dependent variable, and the square root of anti-Pfs25 and anti-CSP AU, respectively, were independent variables in the model. In another linear regression analysis, reciprocal of dilution (in a square root scale) from the original pooled serum to the tested IgG in a feeder was utilized as the independent variable, instead of anti-Pfs25 AU. Since the ratio of anti-Pfs25-specific IgG to entire IgG in a sample is considered to be stable before (i.e., in the original pooled serum) and after (i.e., in the purified IgG) protein G affinity purification, the anti-Pfs25 AU in the original pooled serum and in the purified IgG were used to calculate the dilution factor of each test IgG at each test concentration. Differences within the linear regression analyses were determined using Tukeys HSD.

Statistical tests were performed using Prism 6 (GraphPad Software Inc, United States), JMP11 (SAS Institute Inc, United States) or R (version 3.4.1). *P* < 0.05 were considered significant.

## Data Availability Statement

The raw data supporting the conclusions of this manuscript will be made available by the authors, without undue reservation, to any qualified researcher.

## Author Contributions

FB and SB: conceived and planned the study and wrote the manuscript. YL: prepared Pfs25-IMX313 vaccine for immunization. FB: performed mouse experiments. FB, IT, and AM: performed ELISAs. KM and CL: performed SMFAs. AMS: generated parasites for the ISI assay. FB and AJS: conducted the ISI assay. KM and FB: performed statistical analyses of the SMFA and ISI results. All authors read and commented on the manuscript.

### Conflict of Interest Statement

The authors declare that the research was conducted in the absence of any commercial or financial relationships that could be construed as a potential conflict of interest.
